# Supplementary material for: Modeling glioblastoma heterogeneity as a dynamic network of cell states
Source: Mol Syst Biol. 2021 Sep 16;17(9):e10105. doi: 10.15252/msb.202010105 (PMC8444284; doi:10.15252/msb.202010105)
Supplement: Supplementary file 6 — Source Data for Figure 5 [file MSB-17-e10105-s004.zip › Figure5A_sourcedata/GSEA_3071/nosigdb_stateVI.GseaPreranked.1622795169056/LARSSON_STATE_6.html]

Details for gene set LARSSON\_STATE\_6[GSEA]

|  || Dataset | state63071 |
| Phenotype | NoPhenotypeAvailable |
| Upregulated in class | na\_neg |
| GeneSet | LARSSON\_STATE\_6 |
| Enrichment Score (ES) | -0.6354729 |
| Normalized Enrichment Score (NES) | -2.824976 |
| Nominal p-value | 0.0 |
| FDR q-value | 0.0 |
| FWER p-Value | 0.0 |
Table: GSEA Results Summary

  

Fig 1: Enrichment plot: LARSSON\_STATE\_6      
 Profile of the Running ES Score & Positions of GeneSet Members on the Rank Ordered List

  

| PROBE | GENE SYMBOL | GENE\_TITLE | RANK IN GENE LIST | RANK METRIC SCORE | RUNNING ES | CORE ENRICHMENT || 1 | HOPX |  |  | 89 | 0.761 | -0.0080 | No |
| 2 | TXNIP |  |  | 402 | 0.487 | -0.0842 | No |
| 3 | C4orf48 |  |  | 1297 | 0.303 | -0.3269 | No |
| 4 | ID3 |  |  | 1835 | -0.254 | -0.4711 | No |
| 5 | SAT1 |  |  | 1952 | -0.271 | -0.4975 | No |
| 6 | SNX3 |  |  | 2037 | -0.283 | -0.5146 | No |
| 7 | FYN |  |  | 2118 | -0.301 | -0.5303 | No |
| 8 | MARCH2 |  |  | 2187 | -0.313 | -0.5423 | No |
| 9 | NEAT1 |  |  | 2208 | -0.316 | -0.5409 | No |
| 10 | MXD4 |  |  | 2312 | -0.335 | -0.5622 | No |
| 11 | CITED1 |  |  | 2518 | -0.382 | -0.6109 | No |
| 12 | CTNNA1 |  |  | 2544 | -0.387 | -0.6093 | No |
| 13 | RTL8C |  |  | 2580 | -0.395 | -0.6103 | No |
| 14 | ZMAT3 |  |  | 2590 | -0.399 | -0.6040 | No |
| 15 | FAM20C |  |  | 2610 | -0.403 | -0.6004 | No |
| 16 | METRN |  |  | 2617 | -0.404 | -0.5931 | No |
| 17 | BEX3 |  |  | 2753 | -0.442 | -0.6210 | No |
| 18 | RAB31 |  |  | 2806 | -0.459 | -0.6253 | Yes |
| 19 | PLEKHB1 |  |  | 2810 | -0.461 | -0.6159 | Yes |
| 20 | GABARAPL2 |  |  | 2812 | -0.461 | -0.6060 | Yes |
| 21 | QKI |  |  | 2844 | -0.474 | -0.6041 | Yes |
| 22 | FTH1 |  |  | 2871 | -0.484 | -0.6006 | Yes |
| 23 | MARCKSL1 |  |  | 2928 | -0.506 | -0.6051 | Yes |
| 24 | C1orf122 |  |  | 2977 | -0.528 | -0.6067 | Yes |
| 25 | MAP1LC3A |  |  | 2994 | -0.538 | -0.5993 | Yes |
| 26 | PTMS |  |  | 3004 | -0.545 | -0.5897 | Yes |
| 27 | ID2 |  |  | 3026 | -0.554 | -0.5833 | Yes |
| 28 | FOXO3 |  |  | 3053 | -0.564 | -0.5781 | Yes |
| 29 | PNRC1 |  |  | 3055 | -0.566 | -0.5658 | Yes |
| 30 | SOX4 |  |  | 3066 | -0.571 | -0.5559 | Yes |
| 31 | FABP5 |  |  | 3079 | -0.577 | -0.5465 | Yes |
| 32 | SH3BGRL |  |  | 3095 | -0.582 | -0.5377 | Yes |
| 33 | TNFRSF21 |  |  | 3125 | -0.600 | -0.5325 | Yes |
| 34 | OLFM2 |  |  | 3176 | -0.641 | -0.5323 | Yes |
| 35 | GNAI2 |  |  | 3183 | -0.647 | -0.5196 | Yes |
| 36 | PBX1 |  |  | 3200 | -0.664 | -0.5094 | Yes |
| 37 | PFN2 |  |  | 3215 | -0.685 | -0.4981 | Yes |
| 38 | SOX9 |  |  | 3280 | -0.739 | -0.4996 | Yes |
| 39 | SESN3 |  |  | 3311 | -0.775 | -0.4908 | Yes |
| 40 | RND2 |  |  | 3330 | -0.795 | -0.4782 | Yes |
| 41 | C9orf16 |  |  | 3367 | -0.836 | -0.4697 | Yes |
| 42 | CTXN1 |  |  | 3381 | -0.860 | -0.4542 | Yes |
| 43 | FEZ1 |  |  | 3423 | -0.945 | -0.4447 | Yes |
| 44 | TUBA1A |  |  | 3447 | -1.002 | -0.4289 | Yes |
| 45 | NFIB |  |  | 3508 | -1.145 | -0.4203 | Yes |
| 46 | C1QL1 |  |  | 3520 | -1.186 | -0.3970 | Yes |
| 47 | TUBB2B |  |  | 3532 | -1.228 | -0.3729 | Yes |
| 48 | ZNF428 |  |  | 3558 | -1.402 | -0.3488 | Yes |
| 49 | CLU |  |  | 3601 | -1.801 | -0.3206 | Yes |
| 50 | BEX1 |  |  | 3608 | -1.883 | -0.2805 | Yes |
| 51 | GFAP |  |  | 3619 | -2.337 | -0.2314 | Yes |
| 52 | PMP2 |  |  | 3622 | -2.497 | -0.1766 | Yes |
| 53 | S100B |  |  | 3624 | -2.502 | -0.1214 | Yes |
| 54 | FABP7 |  |  | 3629 | -2.788 | -0.0607 | Yes |
| 55 | CRYAB |  |  | 3633 | -2.841 | 0.0014 | Yes |
Table: GSEA details [plain text format]

  

Fig 2: LARSSON\_STATE\_6: Random ES distribution      
 Gene set null distribution of ES for **LARSSON\_STATE\_6**
